# Priority setting in the Brazilian emergency medical service: a multi-criteria decision analysis (MCDA)

**DOI:** 10.1186/s12911-021-01503-z

**Published:** 2021-05-06

**Authors:** Talita D. C. Frazão, Ana F. A. dos Santos, Deyse G. G. Camilo, João Florêncio da Costa Júnior, Ricardo P. de Souza

**Affiliations:** Departamento de Engenharia de Produção, Centro de Tecnologia, Universidade Federal do Rio Grande do Norte, Natal, 59072-970 Brazil

**Keywords:** Mlticriteria decision analysis, Emergency medical service, Prioritizing victims, Prioritization criteria, FITradeoff, Shortage of resources

## Abstract

**Background:**

Despite the proven value of multicriteria decision analysis in the health field, there is a lack of studies focused on prioritising victims in the Emergency Medical Service, EMS. With this, and knowing that the decision maker needs a direction on which choice may be the most appropriate, based on different and often conflicting criteria. The current work developed a new model for prioritizing victims of SAMU/192, based on the multicriteria decision methodology, taking into account the scarcity of resources.

**Methods:**

An expert panel and a discussion group were formed, which defined the limits of the problem, and identified the evaluation criteria for choosing a victim, amongst four alternatives illustrated from hypothetical scenarios of emergency situations—clinical and traumatic diseases of absolute priority. For prioritization, an additive mathematical method was used that aggregates criteria in a flexible and interactive version, FITradeoff.

**Results:**

The structuring of the problem led the researchers to identify twenty-five evaluation criteria, amongst which ten were essential to guide decisions. As a result, in the simulation of prioritization of four requesting victims in view of the availability of only one ambulance, the proposed model supported the decision by suggesting the prioritization of one of the victims.

**Conclusions:**

This work contributed to the prioritization of victims using multicriteria decision support methodology. Selecting and weighing the criteria in this study indicated that the protocols that guide regulatory physicians do not consider all the criteria for prioritizing victims in an environment of scarcity of resources. Finally, the proposed model can support crucial decision based on a rational and transparent decision-making process that can be applied in other EMS.

**Supplementary Information:**

The online version contains supplementary material available at 10.1186/s12911-021-01503-z.

## Background

Multicriteria Decision Analysis, MCDA is a tool capable of supporting decisions utilizing multiple criteria. The use of MCDA as a method to support the definition of priorities in health care is not new, researchers have shown its growing importance in the health field [1–4] especially within public health systems [5–8]; including recent studies that highlight the contribution of MCDA in the context of epidemic events such as COVID-19 [9–15]. The potential for the application of MCDA in the health field is due to the combination of restricted resources and the growing demands that have led decision-makers to address this issue more directly than in the past [16].

Despite the proven value of MCDA’s support in the health field, no models were found that used a multicriteria method to assist in prioritizing victims’ decisions at Emergency Medical Services, EMS. This is one of the most important health services, as it plays a vital role in saving people’s lives and reducing the rate of mortality and morbidity [17].

Despite the fact that the importance and sensitivity of decision-making in the field of EMS has been recognized by Operations Research scientists, emergency medical planners and health professionals who have studied strategic, tactical and operational problems for EMS since the 1960s [18], only monitoring, forecasting and location problems have been widely discussed [19–23]. Carvalho et al. [24] presents two generic approaches to optimize dispatch; and Belanger et al. [25] relocation decisions and thereby maximize the preparation of the system and a recursive simulation-optimization framework; however, it does not take into account fundamental criteria for the ambulance dispatch decision, such as those related to the victim’s severity and conditions, leaving a gap in the literature.

In Brazil, the solution for these problems is still a manual task, with responsibility for the dispatch decision attributed to the regulating physicians of the Mobile Emergency Care Service, SAMU/192, which is the mobile prehospital component of the Emergency Care System and Brazilian emergencies, in municipalities and regions throughout the national territory. The model proposed by the present work supports the decision to prioritize victims of SAMU/192 regulating doctors, and focuses on the so-called level 1 (red code of absolute priority), since they have the highest number of occurrences. Thus, throughout the text we will use SAMU/192 as a term equivalent to the Brazilian EMS.

The SAMU/192 strategy for sending ambulances follows protocols that are formulated based on criteria related to the victim’s severity level. Based on this information, generally, the victim who is closest to the available ambulance is treated. However, in an environment in which the demand exceeds the capacity of the available resources, a greatest number of attributes related to the alternatives ought to be evaluated; given that from a system overview, this decision can lead to a better area coverage, considering not only the immediate situation, but also possible future emergencies.

For instance, on considering two calls, in a scenario of scarcity of resources, in which the victims suffer from epileptic attacks [26, 27]; after assessing the victim’s health status, the regulatory physician (RP) classifies the call as Level 1, red code and authorizes the ambulance to be dispatched. However, only one ambulance is available to assist the victims. Given this scenario, when the regulatory protocols are not sufficient for the decision to prioritize victims, how to make the decision? What are the steps to be followed? Which victim to prioritize? When the protocol advises the ambulance to be sent and the doctor does not have the resources to care for the victims, what criteria would be used to assist this decision? The prioritization of victims must be carried out, making use of guiding criteria (protocols - dealing with criteria related to the victim’s health status) and criteria that influence the decision-making process (criteria related to the health system; support tools; victims’ details and external factors).

In such cases, which involve different criteria, the use of the multicriteria model developed will bring greater clarity, transparency and rationality, maximizing the benefit and minimizing the risk in the SAMU/192 environment. Hence, the current study presented the dispatch problem as a decision to prioritize victims and developed a new model for prioritizing victims of SAMU/192, in an environment of scarcity of resources based on multicriteria decision support methodology. The analysis and structuring of the problem revealed important criteria in the field of EMS. Mathematical modeling, with FITradeoff, suggested the best alternative amongst those analysed. It is believed that the proposed model could improve the efficiency of victim prioritization in the context of SAMU/192.

### Multicriteria decision

A multicriteria decision problem occurs when the Decision Maker, DM, an individual over whom power and responsibility over the decision are attributed, is faced with a situation with at least two alternatives for action, so that choice between the courses of action available is driven by the desire to meet multiple objectives, conflicting with each other [28–30].

Before selecting and implementing any MCDA method, the limits of the problem to be addressed should be defined, given that the better the problem is defined, the more accurate the analysis result will be [31, 32]. This can be achieved by reviewing the literature on decision-making criteria, conducting qualitative research, and consulting experts [3].

As long as the problem was pre-defined, the objectives and criteria to assist in decision making were identified, and the decision maker’s rationality established, a multicriteria assessment method can be chosen to meet the conditions and needs to address the problem [29, 33].

### Multicriteria model of deterministic additive aggregation

An additive model for aggregation uses a single synthesis criterion MCDA method, which presents as the main characteristic the aggregation of multiple criteria in a single synthesis [29], thus being situated in the context of compensatory rationality. In such model, uncertainty is considered in obtaining the vector of consequences *x* for each alternative *a*.

For the problem of choice, which defines the class of problems in which the objective is to support the decision through the choice of a subset of the action space, the solution of the additive model consists in the selection of the alternative that presents the highest global value *v*(*a*) [30]. As the contribution of the additive model to the single criteria methods of synthesis lies in the process of aggregating the criteria, in these methods the evaluation of the alternatives is carried out through the value function defined on the consequences, considering that each alternative is associated with a consequence vector *x* [29].

The greatest difficulty faced by the multicriteria decision support methodology lies in the evaluation and modelling of preferences [30]. However, preferences can be modelled by rules and logical relations [34–36]. With the knowledge about the preferences of the decision maker, a problem can be solved based on the additive model, being necessary two types of evaluation: the intracriteria and the intercriteria. The intracriteria evaluation aims to evaluate each alternative *i* for each criterion *j*, which leads to the value function $v_j(a_i)$, the construction of the value function for each element is based on the evaluation of the consequences to be obtained. In the Intercriteria evaluation with the information $v_j(a_i)$, the information that considers the combination of the different criteria is sought, and to find it, it is necessary to choose a method of aggregating criteria [29]. When the criteria are represented by different units, it is necessary to normalize these values, so that they are redefined on a scale from 0 to 1. Prior to the final recommendation, sensitivity analyses can be carried out to investigate whether the preliminary conclusions are robust or whether they are sensitive to changes in aspects of the model [32].

### FITradeoff

Almeida et al. [37] proposed the Flexible and Interactive Tradeoff, FITradeoff method to address the problem of eliciting scale constants, which will occasionally be referenced also by criteria weights. In FITradeoff the scaling of the scale constants is based on the tradeoff procedure in which the values tradeoff are to be achieved, which is defined by the moment when the decision maker is indifferent to two consequences and you can be willing to exchange them [29, 37]. This procedure is adopted in the Tradeoff method [38, 39], which makes FITradeoff to be considered an extension of this.

In compensatory methods, the DM considers the compensations by criterion when comparing the alternative consequences [38, 39]. Operationalization through the Decision Support System (DSS), includes the following steps: 1. Intracriteria assessment; 2. Classification of criteria weights; 2.1. Attempt to solve the problem using the set of weights available; 3. The DM’s preferences are evaluated to arrive at the results [37].

The first part allows the weight space to be defined. Subsequently, the second part is started, it is possible to see the difference between the procedure and the traditional model, the DM is not required to define an exact value of ${(x_{l}^{i})}$, which denotes the result of the *i* criterion for which indifference is obtained between consequences, whereas the traditional method requires this procedure.

If the solution is not found, then the third stage begins, that is, that of assessing the preferences of the DM that can be divided into four stages: 3.1. Define values to test the weight distribution; 3.2. Asking the DM to indicate their preferences; 3.3. LPP computing; and 3.4. Finalization. Upon completion, these four steps constitute the main stage of FITradeoff. Thus, the objective is to find an alternative, based on the vector of the alternatives, which has the maximum value according to the weight of the criteria space [40]. Therefore, LPP is performed until an ideal alternative is found. If it does not occur, the dominated alternatives are eliminated and the process is started again, starting from step 3.1. Now, only the alternatives identified as potentially optimal are considered in the subsequent steps, otherwise the process is finalized [37]. In the finalization step, the weight ranges supporting the solution are computed and produced in a report with the final recommendation.

## Methods

### Study location and decision-making actors

The current research is based on the specialized knowledge of representatives of the nine states of the Northeast Region of Brazil, which is composed of nine states: Alagoas, Bahia, Ceará, Maranhão, Paraíba, Pernambuco, Piauí, Rio Grande do Norte and Sergipe. An expert panel was formed by fourteen medical regulators who contributed to the structuring of the decision problem through semi-structured questionnaires. Carrying out the structuring phase with a varied group of stakeholders, makes the process more robust, as it provides a common view of the problem and allows the integration of different perspectives [41].

The observations and discussion meetings took place at SAMU/Natal in Rio Grande do Norte, RN, with a discussion group formed by the General Director, the Medical Coordinator, the Regulation Coordinator and one of the most experienced on-call chiefs at the regulation centre. Everyone could express their preferences in relation to the cases presented. The decision maker appointed was the General Director of SAMU/Natal-RN, who is also a regulatory physician. An analyst mediated the decision-making process, thus making unnecessary to use techniques to reach a consensual position amongst the group members. Other stakeholders were selected based on simultaneous participation throughout the study.

### Regulatory physicians’ general decision aspects

Throughout the development of the multicriteria decision model, the entire path from the call to the victim’s transport to a health unit was completely mapped, as no information important to the decision, which occurs in real time, could be disregarded.

**Fig. 1:**
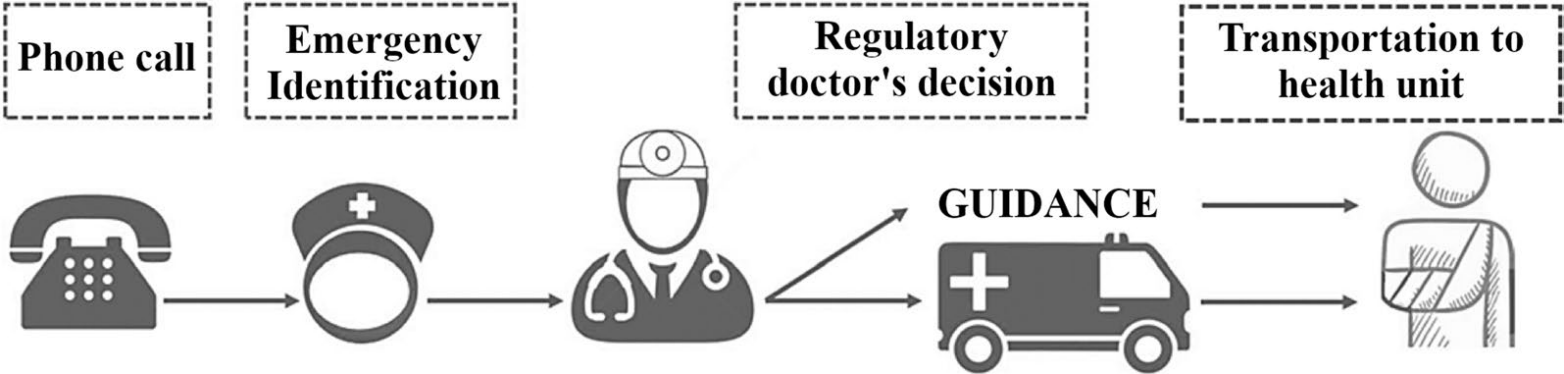
The operation of SAMU/192

Assistance to victims begins with the telephone call, when guidance is provided on the first actions. The call is answered by medical regulation assistant technicians “MRAT” who identify the emergency and collect the first information about the victims and their location [42]. After the reception and identification of the calls, requests are judged by the Regulatory Physician, RP, who classifies the level of urgency of each call, defining the necessary resource for their adequate attendance, which can involve from a simple medical advice to the activation of a Radio Operator, RO, who will activate the nearest or most appropriate ambulance, depending on the severity of the situation. After the on-site assessment, the patient will be transported safely to the health services. As shown in Fig. 1.

SAMU functioning is presented on Fig. 1.

The current research follows three main phases: the decision problem is structured and, after selecting the MCDA method, preference modelling is carried out.

### Structuring the decision problem

#### Problem, objective and alternatives of the MCDA model

Two meetings were held at the SAMU/192 Natal Regulation Centre within a six months interval “April to October 2018” with a discussion group. As a result, the delimitation of the decision problem began, definition of the panel of experts and the alternatives were established as: (1) four victims, and (2) four cases (two clinical and two traumas) were selected, classified as Level 1 , red code. The choice of topics was based on the SAMU/192 service history. When analysing a sample of historical data from 2015 to 2018, chosen for convenience, the results revealed the clinical and trauma cases as the most representative specialties, as together they account for 84.77 % of the total number of diagnoses. The cases were prepared based on the literature and on the experience of the regulating physicians at the Natal-RN Regulation Centre [43].

#### Rating criteria

To identify the evaluation criteria, a mainly qualitative approach was chosen as being more appropriate to the exploratory nature of the study and to address the research question, maximizing credibility, reliability and the findings confirmation. The qualitative research instruments that were employed in this phase included observations, document review, open and semi-structured interviews. The combination of such instruments enabled the most complete and multifaceted examination [44].

*Observations and open interviews* The non-participant field observation of the daily work practices of the professionals at the SAMU/Natal-RN regulation centre had a greater focus on Regulatory Physicians, RPs. The objective of this initial stage of data collection was to gain an understanding of the organizational context and the decision making of the RPs in practice.

The observation involved conducting open interviews with a number of key Regulatory Central employees (n = 30) to provide an overview of the organization and service delivery (between December 2018 and June 2019). Such individuals included coordinators and medical regulators, whose functions alternated between operational and leadership management. Although the character of the observations was non-participant, the open interviews aimed to capture internal and external perspectives on regulation and systemic influences [45].

Throughout the open interviews it was collected: (1) key demographic information; (2) relevant documentation dealing with policies and procedures; and (3) the empirical experience of the of EMSs victims’ prioritization decision. The data collected in the observations and open interviews were part of the necessary framework for the elaboration of the semi-structured interview, allowing the identification of twenty-one decision criteria that were categorized in five dimensions: (1) health system; (2) healthcare tools; (3) victims’ profile; (4) victim’s health status; and (5) external factors.

*Document review* The protocols for basic and advanced life support used in the context of the organizational environment were examined [46, 47] together with the pre-established criteria by the support system of the central regulation, the latter being used for the investigation of the presumed severity, during the call. With the analysis of such documents, four of the six criteria related to the victim’s health were extracted. At the end of this stage, amongst the data collected, there were twenty-five decision criteria for the problem of prioritizing victims, subdivided into five dimensions. These data were presented to the discussion group, in a meeting, in which it was agreed that such criteria would undergo a review and a second validation.

*Semi-structured interviews* The final step in selecting the criteria involved conducting semi-structured interviews with SAMU representatives from the nine states in the Northeast Region in Brazil. An online questionnaire was developed based on the data collected in the observation process, in the open interviews and in the document review (see Additional file 1). A total of fourteen medical specialists participated; amongst them eleven were in leadership positions and had between five to ten years of experience in emergency medical regulation.

Regarding the questionnaire, the presentation of the questions was preceded by the central problem presentation to the participants: a scenario in which doctors receive three calls with the same characteristics, three victims who complain of pain, and to answer them they have only one ambulance is available. There was also the observation that cases should be treated as an urgent priority. After this introduction, doctors were presented with two questions, one that presented the criteria and another discursive question. The first questions stated: What do you need to know to make the decision? What can influence this decision, knowing the protocol advises the dispatch, even though resources are scarce? Mark all the options you need to make this decision, check as many as needed. The second questions asked: At the time of the decision, based on your experience and skills, could any other criterion or dimension not present in the protocols or in this questionnaire be added?

### MCDA method selection

For the multicriteria method selection, the basic preference situations were observed, mainly to support the decision maker’s preference modelling, as well as the rationality considered by him to the context under study [36]. The decision support system (DSS) that accompanies FITradeoff was also an important aspect for the selection, as it was built under a concept of flexible elicitation that requires less cognitive effort from the decision-maker because it requires reduced information, without the need for indifference adjustments between its consequences. Thus, it becomes easier for the decision maker to make comparisons of results based on strict preferences and not on indifference [37].

### Modelling preferences

Once the criteria list was defined as well as their corresponding scale constants, the intracriteria evaluation was performed, obtaining the functions of value $v_i(x_i)$ for each criterion *i* which, in turn, enabled the construction of the decision matrix. Afterwards, the data from the previous stage were analysed in a discussion group meeting; the decision maker’s preference was considered linear for all criteria [48]. For the standardization procedure applied in this work, the scale is from 0 to 1, in which 0 and 1 represent, respectively, the minimum and maximum values of the performances in the criterion [37]. The intercriteria evaluation was carried out using the FITradeoff multicriteria method, in the FITradeoff *software* for the additive model of choice with sensitivity analysis, (FU-T1EEMO-CT1).

Sensitivity analyses were also performed with all the criteria in order to examine the robustness of the selection and to identify possible weight sensitive criteria. The percentage values for variation of the criteria were $-20$% and +20%. The consequence value of each alternative *j* of the problem for the criterion *i* assumed a value *Sj*, *i* such that $\left(pj,i\right)\ast \left ( 0,8 \right)\leq Sj,i\leq \left ( pj,i \right)\ast \left ( 1,2 \right )$, where *pj*, *i* corresponds to its nominal value (value of the original consequence matrix). To maintain the original limits of the consequence space, the SAD verifies the minimum value of the consequence space generated in the Sensitivity Analysis cycle and assigns the minimum nominal value to it. The same is done for the maximum nominal value.

## Results

### Structuring the decision problem

#### Problem, objective and the MCDA model alternatives

Based on the objective of prioritizing victims, the main issue is the choice of which victim to attend first. To this end, the alternatives presented were illustrated based on four occurrence scenarios that brought physicians the main points of pre-hospital care in emergency situations and clinical and traumatic emergencies, these classified as emergencies of absolute priority. The Table 1 presents description of the alternatives presented to the professionals.

The description of the alternatives presented can be seen in Table 1.

**Table 1:** Description of the alternatives

**Alternatives**		
*Information*	*Victim 1*	*Victim 2*
Case	Call to the 192 emergency number answered by the medical regulation assistant technician (MRAT). Reason for the call: “tightness in the chest and difficulty breathing” . The caller for the mobile emergency care service (SAMU), the victim’s co-worker, told the MRAT that a 22-year-old military firefighter was attacked by a swarm of African bees. The accident occurred when he was carrying out a training exercise in a region far from the municipality. At the time of the request, the victim had hives, itching and chest discomfort associated with sudden onset severe dyspnoea, at the time of the occurrence it started raining on the spot, the applicant insistently warned the regulatory physician that the victim was allergic to bee sting. The ambulance dispatch was authorized by the doctor to the advanced support unit (ASU). Diagnostic Hypothesis: anaphylaxis /poisoning syndrome	Call to the 192 emergency number answered by the medical regulation assistant technician (MRAT). Reason for the call: motorcycle-bicycle collision. A passer-by triggers the mobile emergency service (SAMU) reporting to the MRAT that he witnessed a motorcycle-bicycle collision, leaving a male victim, apparently aged 40, breathing but unconscious, and bleeding profusely. To the regulator, the applicant reported that the victim was riding the motorcycle, without a helmet, and that, at the moment, he was not responsive and had severe bleeding from the nose and ear. The Advanced Support Unit was authorized. Diagnostic Hypothesis: severe traumatic brain injury
Victim’s location	Pajuçara—12,4 km of the driving distance—30 min	Lagoa Azul—12,1 km of the driving distance—27 min
Location’s Characteristcs	Traffic congestion; native vegetation area; no paving; raining; there is insistence by people close to the victim	Traffic congestion; duplicate road; on the road; high incidence of sunlight; agglomeration of people
*Information*	*Victim 3*	*Victim 4*
Case	Call to the 192 emergency number answered by the medical regulation assistant Technician (MRAT). Reason for the call: motorcycle fall. The caller reaches the mobile emergency service (SAMU) to assist a 23-year-old victim of a motorcycle fall, who was conscious, breathing and bleeding profusely. To the regulator, he reported that the victim was driving the vehicle without wearing a helmet, lost consciousness at the time of the accident and had an apparently severe head injury. The Basic Support Unit was sent for the location. During the treatment, the patient developed an episode of post-traumatic tonic-clonic seizure crisis. The Regulatory Physician then authorized the Advanced Support Unit for the Site. Diagnostic Hypothesis: moderate traumatic brain injury, with evolution of tonic-clonic post-traumatic seizure crisis	Call to the 192 emergency number answered by the medical regulation assistant technician (MRAT). Reason for the call: Difficulty breathing. The applicant, a relative of the victim, calls the mobile emergency service (SAMU), and informs the MRAT that a 60-year-old was at home when he felt nauseous with difficulty breathing. He reported to the regulator that the victim suffered from hypertension, heart disease and was presenting sudden dyspnoea followed by syncope and decreased level of consciousness. The applicant also reported that the pulse was still palpable, but very weak. Diagnostic Hypothesis: Respiratory insufficiency, plus a decrease in the level of consciousness
Victim’s location	Nossa Senhora da Apresentação—118 km of the driving distance—28 min	Ponta Negra Village—12 km of the driving distance—30 min
Location’s Characteristcs	Traffic congestion; narrow streets; Victim is on the road exposed to change of climate	Heavy traffic; duplicate road; victim is home; there is insistence by relatives

#### Rating criteria

After the observation period, open interviews and document review, it was concluded that the information investigated by the regulating physicians with the potential to influence at the time of the call may be of the following different types:

1. Criteria related to the Health System: access to the doctor, access to the bed, and support from security agencies;
2. Criteria related to Support Tools: location of the ambulance, access to the assistance system, access to support material;
3. Criteria related to the Victim: location of the victim, proper means of transportation, age, time of call, health insurance, sex, refusal, willingness;
4. Criteria related to the Victim’s Health Status: airway and oxygenation, state of consciousness, pulse, traumatization, victim’s health history, alcohol or drug intake;
5. Criteria related to External Factors: road traffic congestion; social commotion; bad weather, hard to reach address, insistence of people close to the victim.

**Fig. 2:**
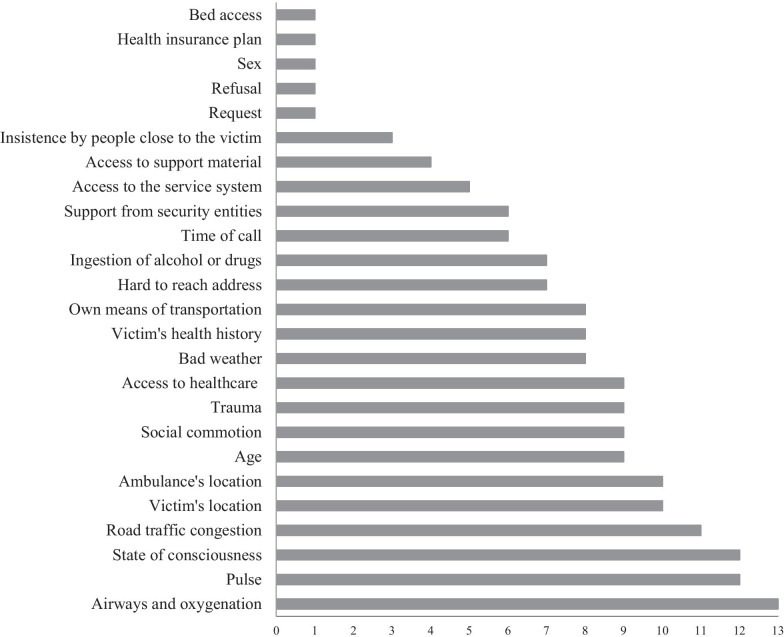
Decision criteria assessed by the expert group

Decision criteria evaluated by the group of experts, of the semi-structured interviews, is presented in Fig. 2.

The results of the semi-structured interviews sent to the panel of experts are presented in Fig. 2, in which it can be observed that the three main ones are related to the victim’s health status, followed by access criteria and the victim’s location. Willingness, refusal, sex, health insurance and access to the bed, in most cases, are not priority information for specialists. The other criteria can be considered important in a decision to prioritize victims as the doctors have shown interest. The answers presented in the second question indicate that the experts agreed with the classes of criteria presented and did not add any other to the list.

#### Selected criteria

For the cases under evaluation, the discussion group agreed that the list would be complete if it contemplated the ten criteria described in the Tables 2 and 3.

The final list of criteria related to the victim’s health is shown in Table 2.

**Table 2:** Final list of criteria related to the victim’s health

Dimension	Criteria	Description	Scale
Criteria related to the victim’s health	Airways and oxygenation	The victim breathes normally, does not breathe or has a loud or altered breathing. This has a direct impact on the victim’s health	They’re on a verbal scale, ranging from one to four. Where 1 stands for Unhurt, and 4 Severe (Unhurt, Small, Medium and Severe). A higher value is preferable to a lower value. (Maximization)
	State of consciousness	The victim seems normal, confused, unconscious, had a seizure or loosened sphincters	
	Pulse	Investigation of the victim’s pulse, the doctor wants to investigate if it’s normal, fast, slow or non existent	
	Airways and oxygenation	The victim breathes normally, does not breathe or has a loud or altered breathing. This has a direct impact on the victim’s health	
	Trauma	The doctor wants to know if there’s visible bleeding, body deformity, burns, incarceration or confinement	
	Victim’s health history	Historical data of the ailment, recurrence, hereditary diseases, or any other information that might contribute to the treatment	

Access to the service system and support material was considered to be available and would not influence the decision. Regarding the time of the requests, it was pre-established that the calls were registered in the same time interval. Finally, with regard to willingness and refusal (to be attended), it was considered that both the family and the victim wanted care. The location of the ambulance, the intake of alcohol or drugs, the support of security agencies, access to the bed and the sex of the victim were not considered for the evaluation of the four applicants as the discussion group would not find these to be relevant criteria for the cases.

The final list of criteria related to the victim and external factors is shown in Table 3.

**Table 3:** Final list of criteria related to the victim and external factors

Dimension	Criteria	Description	Scale
Criteria related to the victim	Age	Victim’s Age.	It’s represented by the age of the victim. A lower value is preferable to a higher value. (Minimization)
	Location	The location of the victim, the distance from the relief facility.	It’s represented by the distance from the victim to the ambulance, expressed in Km. A smaller value is preferable to a higher value. (Minimization)
	Access to healthcare	If the victim has a health plan, if the victim has and is able to be removed to a medical facility by their own means.	The measurement is a verbal scale, ranging from 1 to 4. Where 1 means very bad and 4 means very good (very bad, bad, good, very good). A lower value is preferable to a higher value. (Minimization)
Criteria related to external factors	Access to victim’s location	If the address is in rural areas, unpaved, areas with greater presence of vegetation, or regions with high crime rate. If there’s slow traffic or congestion	
	Social commotion	It would be the discontent or outrage resulting from the victim’s situation. If the people close to the victim who are awaiting service insistently return the call to the regulatory center, requesting immediate service. If the victim is exposed to any weather conditions that are more intense	They are on a verbal scale, ranging from 1 to 4. Where 1 means none and 4 means high (none, low, medium, high). A higher value is preferable to a lower value. (Maximization)

The other criteria were included in the model and to facilitate and streamline the decision-making process, those with similarities were grouped: the estimation of access to the victim’s location took into account the difficulties of access created by both roads and vehicles (road traffic congestion + difficulty to reach address), the social commotion analysed information about popular indignation and the climatic conditions to which the victims were exposed and, finally, regarding the criterion of access to the doctor, the MR evaluated if the victim had any condition that would allow him to have access to the doctor safely (health plan + proper means of transportation). Amongst the ten selected criteria, eight are on a verbal scale ranging from 1 to 4.

**Fig. 3:**
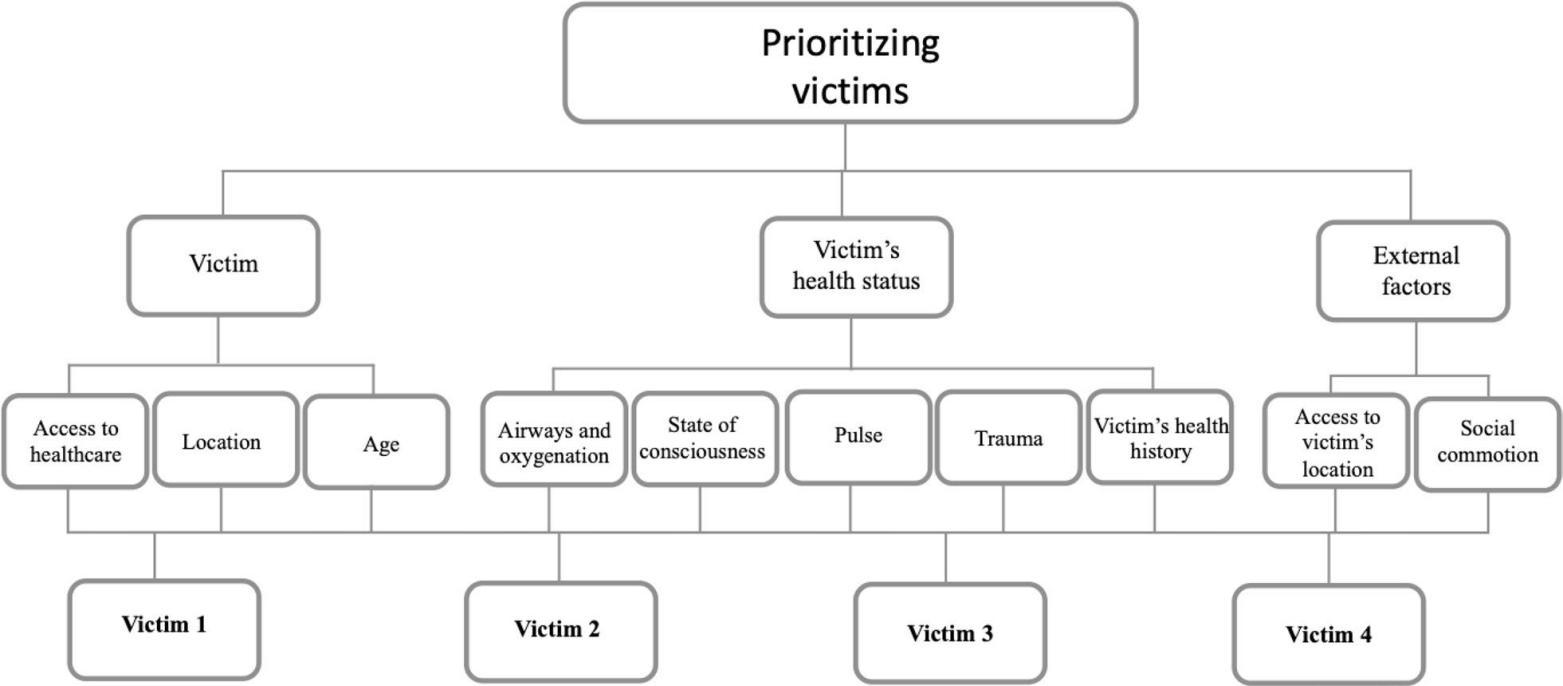
Objective, criteria and alternatives of the proposed model

The objective, criteria and alternatives of the proposed model are presented in Fig. 3.

The Fig. 3 presents the summary of the results found so far: the objective of the decision model, the ten criteria and the alternatives.

### MCDA method selected

It was observed that the input information is aligned with the analysed method as the DSS operates with numerical inputs. Furthermore, the decision-maker presented compensatory rationality, in which it is admitted that there may be an absolute compensation between the different evaluations, given that the reality of the structured decision problem, a good performance in ’airways and oxygenation’ can easily counterbalance a poor performance in ’victim’s history’. This rationality profile is in line with that for the application of FITradeoff.

As the decision maker was able to make comparisons between pairs of consequences with strict preference and indifference relations, the preference structure (P,I) was sufficiently adequate for modelling the decision maker’s preferences. As the approach of the single synthesis criteria methods does not accept that there may be good reasons to justify the incomparability between two alternatives, under this circumstance, it was admitted that the alternatives can be compared with each other, therefore, there is no presence of incomparability.

### Modelling preferences

The Table 4 evaluated each alternative *i* for each criterion *j*, which led to a function of value $v_j(a_i)$, corresponding to the intracriteria evaluation. The Table shows the format of the input data for the chosen multicriteria method. It was added information to the data on the classification of the criteria (whether continuous or discrete), the type of function that each criterion will assume. In the case of discrete criteria, it was necessary to inform the number of scale levels to be considered.

The decision matrix is shown in Table 4.

**Table 4:** Decision Matrix

Alternatives	Criteria									
	Victim			Victim’s health status					External factors	
	Location KM	Age	Access to healthcare	Airways and oxygenation	State of consciousness	Pulse	Trauma	Victim’s health history	Access to victim’s location	Social commotion
Victim 1	12.4	22	2	4	1	1	1	4	1	3
Victim 2	12.1	40	1	4	4	4	4	1	3	3
Victim 3	11.8	23	3	3	4	3	4	1	3	2
Victim 4	12	60	2	3	4	2	1	4	2	2

**Fig. 4:**
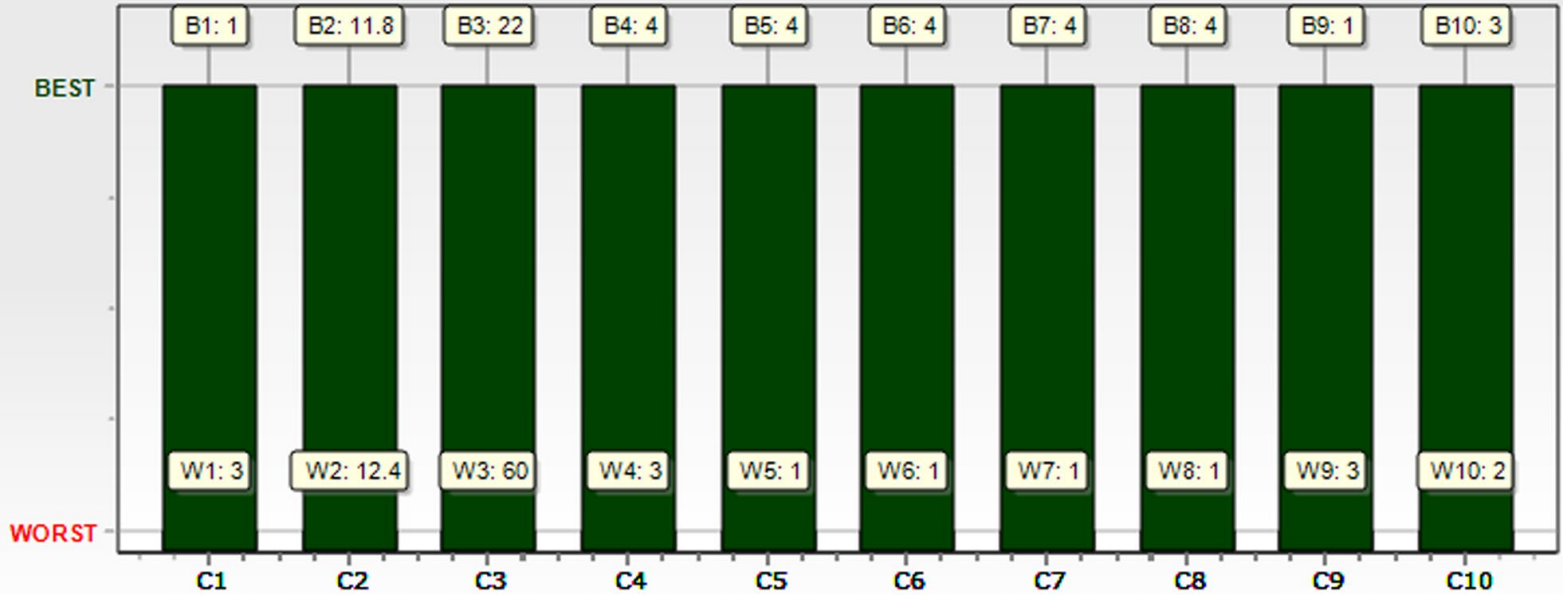
Ordering of the criteria

**Fig. 5:**
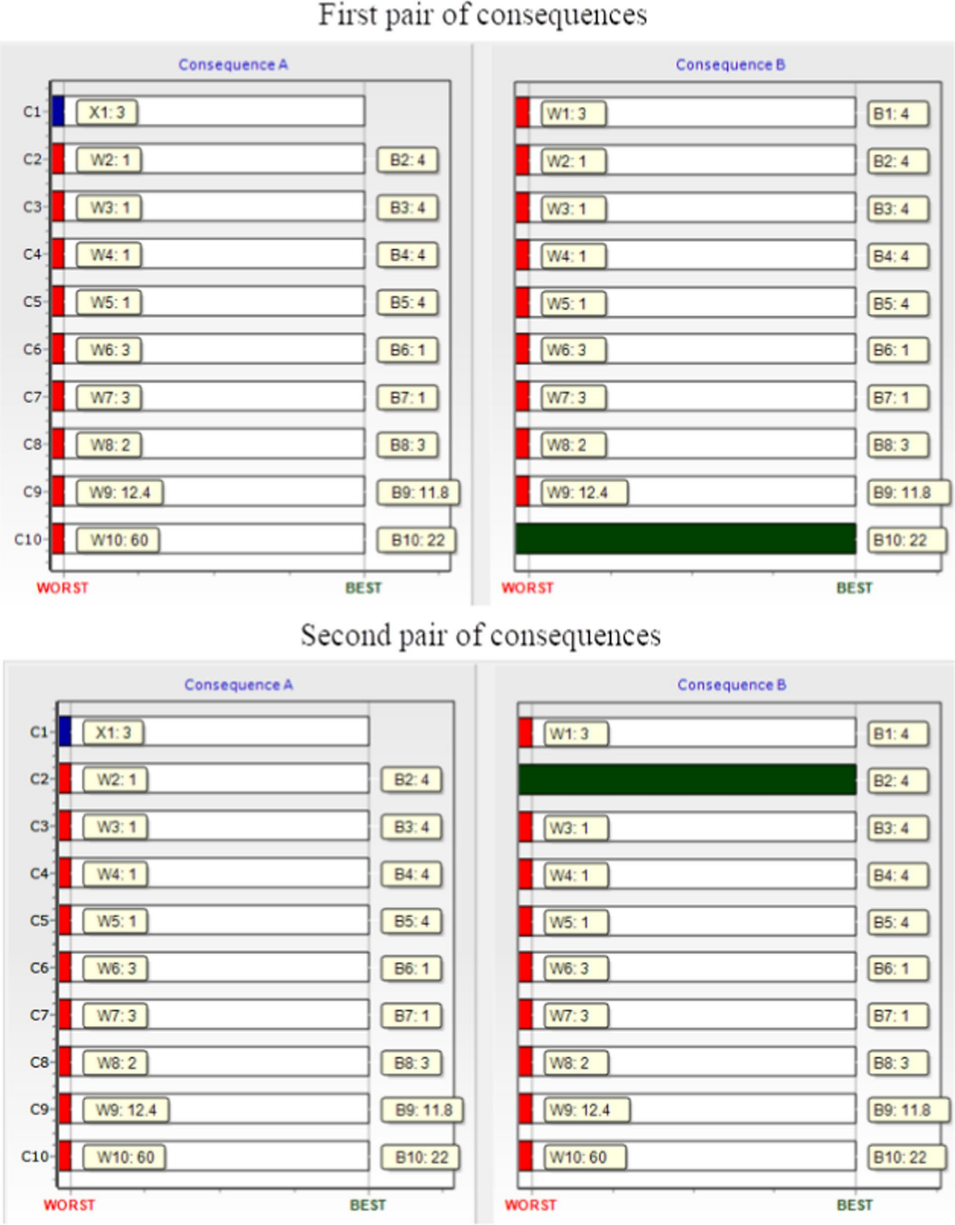
Pairs of consequences and the chosen scenarios

After structuring the problem considering an additive model, the data in the Table 4 were utilized and entered into the DSS. Subsequently, the FITradeoff steps were operationalized. After sorting the criteria (see Fig. 4), flexible elicitation started, and two questions to the decision maker were enough for a single solution to be found for the weight space. Figure 5 shows the two pairs of consequences presented to the decision maker, who in the first question preferred scenario A and in the second, B.

The ordering of the criteria is shown in Fig. 4.

The pairs of consequences and the chosen scenarios are shown in Fig. 5.

**Fig. 6:**
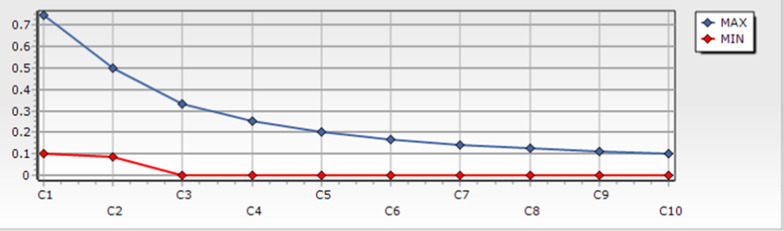
Weight ranges

The recommendation was to prioritize Victim 2. Figure 6 illustrates the departure of the DSS, presenting the space of viable weights for which Victim 2 is pointed out. The breadth of the value range demonstrates the robustness of the result in view of the variety of weights associated with the main criteria, in decreasing order, for which Victim 2 would still be pointed out.

Weight ranges are shown in Fig. 6.

The weight range for each criterion is: Airways and oxygenation (0.75–0.1); State of consciousness (0.5–0.084); Pulse (0.333–0); Traumatization (0.25–0); Victim’s health history (0.2–0); Access to the doctor (0.17–0); Access to the victim’s location (0.14–0); Social commotion (0.13–0); Location (0.11–0); and Age (0.1–0). These weight ranges result from LPP models, considering maximum and minimum weights, subject to restrictions for the development alternatives.

**Fig. 7:**
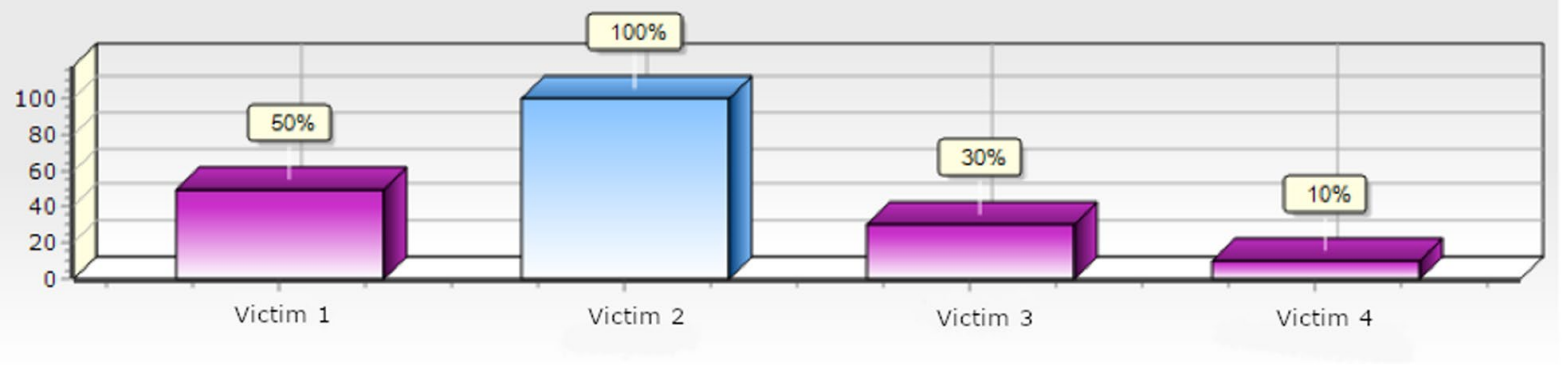
Alternatives found during the execution of the sensitivity analysis

The alternatives found during the performance of the sensitivity analysis are shown in Fig. 7.

The Fig. 7 illustrates a graph with the alternatives that were present in the subset of potentially optimal alternatives during each cycle of Sensitivity Analysis. Among these alternatives, those that were also present in the original subset appear in blue. Those that were present in the subset in some sensitivity analysis cycle, but were not in the nominal subset, are shown in purple. It is also observed the impact of changes in the selected alternative, which suggests that Victim 2 remains consistently the most attractive alternative, whatever the assumed value. These results demonstrate the robustness of the recommendation provided.

## Discussion

The present work has developed a multicriteria decision model to support the decision of the regulatory physicians, considering an environment of resources scarcity, whilst MCDA was not yet utilized to assist this type of decision. As a result of the analyses of the specialist doctors and discussion group, different dimensions of criteria were created, guiding criteria, as well as, those that influence the prioritization of victims were analysed and considered. This knowledge from regulatory protocols, and from the experience of specialists, can support the decisions of regulatory physicians who are still newcomers. The conclusions of Perona et al. [49] corroborate with our findings, as he stated that there is a difference in the processes of thinking and storing information between specialists and novices.

It was also observed that the structuring of the problem facilitated the decision makers’ learning and understanding about the problem faced, about their own priorities, values and objectives of other parties. The exploration of this context was able to guide them in the identification of a course of action, and made it possible to learn about the twenty-five evaluation criteria. However, for prioritization in the four cases presented, only ten would be necessary, which were criteria related to the victim’s health status, the victim’s details and external factors. These criteria do not invalidate the others, as they may be necessary in different contexts, for prioritization, for instance in a psychiatric case, the criterion “support from security agencies” will be part of the decision.

After structuring the decision problem, the specialists analysed four cases. In this phase, the results indicated that the decision maker would be able to identify relations of preference and indifference between the pairs of consequences. In this decision making situation, it was found that the alternatives can be compared with each other, therefore, there is no presence of incomparability. The modelling of preferences performed with the multicriteria FITradeoff method, pointed at victim 2 as the best choice, a result that was supported by experts and decision makers. Thus, the flexible and interactive Tradeoff process proved to be effective for prioritizing victims in this context. This result attested that the model was well structured and that the method was adequate to the problem specificities. Almeida et al. [29] explains that a decision model corresponds to a simplified formal representation of the problem faced, supported by a multicriteria method. Mathematically, the sensitivity analysis showed that Victim 2 has consistently remained the most attractive alternative, whatever the assumed value.

No studies were found using the MCDA to support decisions in this specific area involving the participation of medical specialists. The recent findings in the literature on decision-making in EMS focus on the following points? (1) managing ethical aspects of advance directives in emergency care services; (2) decision support for patient admission; (3) presentation of a structure that provides healthcare professionals with a just-in-time basis; and (4) exploration of the main challenges and barriers that affect clinical decision-making [49–52]. In summary, these studies determine paramedics’ decision-making styles, and also question how decision-making is carried out in this context [53–57].

Other quantitative studies question the idle dispatch policy and propose a multi-verse optimizer algorithm for repositioning ambulances in emergency medical services systems, for which states are defined based only on the locations of idle ambulances waiting at stations, and the occupied ambulance locations were approximated based on customer arrival rates [58, 59]. Furthermore, it could be mentioned other relevant works that seek solutions to localization problems [21–23, 25].

These works focus on a single criterion and/or the methodology used is not adequate to take into account several criteria simultaneously when supporting the decision maker. Their analyses enrich the literature, however, the decision maker needs not only the analysis, but also, a direction on which choice may be the most appropriate, based on different and often conflicting criteria.

The present study has some limitations; for instance, the methodology is validated through only four cases analysis (two clinicians and two trauma victims). As a result, some criteria are left out of the model. New cases must be studied to validate the list of criteria and/or add new ones, necessary for similar or different cases.

Several performance dimensions can be considered when estimating victim prioritization criteria. These criteria may include the location of the ambulance, access to the doctor, the age and state of consciousness of the victim, as well as whether the case is characterized by social commotion. However, in the current study, it was not possible to determine a universal set of victim assessment criteria for a single model because each case has specific performance criteria.

It was also observed that the majority of the evaluation criteria are common to all cases, although they are adapted to the special characteristics of different priorities (for instance, the meaning of access to the doctor may differ if the criterion refers to the health system and not the victim). This common set of criteria allows the development of a uniform evaluation structure as well as additional comparative analyses. However, there are some criteria that differ according to each case. For instance, the support attribute of security agencies would be a priority in cases of psychiatric emergency.

The model, if more evident, can then be used in planning, training and even online devices for decision making in this area. Here, the positive character of the methodology used is emphasized due to the possibility of revising the previous steps before making the recommendation to the decision maker.

Therefore, the flexibility of the MCDA process and the importance of the stage of structuring the problem are highlighted, considering its impact on the successor stages and its critical character regarding the application of the chosen method. In many cases, the efficacy of the multicriteria approach is due precisely to the discussions and a greater degree of understanding of the problem; given that the problem structuring phase has presented to all those involved, criteria unknown to the decision regulation protocols, albeit important for the regulating physicians.

Thus, it is evident that there is a vast field of research exploration for Multicriteria Decision Analysis in prioritizing victims of pre-hospital emergency care, improving the sector. Thus, future research is recommended to explore the efficiency of the model in different scenarios.

## Conclusions

This work contributed significantly to the rational, transparent and impartial practice of prioritizing victims of SAMU/192, using the multicriteria methodology to support the decision making process. After the formalization of the model, reproducibility and validation seem to be possible, which are two important parameters when dealing with the challenge of regulating physicians’ decisions.

Selecting the criteria in the current study led to the realization that the protocols that guide regulatory physicians do not take into account all the criteria for prioritizing victims in an environment of resource scarcity. The structuring of the problem made it possible to learn about twenty-five evaluation criteria. The prioritization of victims must be carried out, using criteria that guide (protocols) and criteria that influence the decision-making process. For mathematical modelling, the flexible and interactive tradeoff process proved effective for prioritizing victims.

Lastly, the current authors sustain, based on the evidence collected, that the developed model has the potential to support the SAMU/192’s regulatory physician to direct resources to victims who need support the most, and can be used in other pre-hospital emergency care units.

## Supplementary Information

**Additional file 1.** Victims Prioritization - Semi-structured interviews.

## Data Availability

The datasets used and/or analysed during the current study are available from the corresponding author on reasonable request.

## Abbreviations

MCDA: Multicriteria decision analysis
EMS: Emergency medical services
Samu/192: Mobile emergency care service
DM: Decision maker
FITradeoff: Flexible and interactive tradeoff method
DSS: Decision support system
RN: Rio Grande do Norte
RPs: Regulating physicians
TARMs: Medical regulation technicians
RO: Radio operator
USA: Advanced support unit
