# Supplementary material for: Priority setting in the Brazilian emergency medical service: a multi-criteria decision analysis (MCDA)
Source: BMC Med Inform Decis Mak. 2021 May 6;21:151. doi: 10.1186/s12911-021-01503-z (PMC8100937; doi:10.1186/s12911-021-01503-z)
Supplement: Supplementary file 1 — Additional file 1. Victims Prioritization - Semi-structured interviews. [file 12911_2021_1503_MOESM1_ESM.pdf]

Victims Prioritization - Semi-structured interviews

Research Objective: To determine the key criteria for victims prioritization, classified as absolute priority, in a situation of resources scarcity.

Decision making circumstances: a scenario in which doctors receive three calls with the same features, three victims who complain of pain, and they have only one ambulance available (one Advanced Support Unit).

Note: Such cases must be treated as Absolute Priority Urgency.

Question 01

What do you need to know to make the decision? What can influence this decision, knowing the protocol advises the dispatch, even though resources are scarce? Mark all the options you need to make this decision, check as many as needed. (**Mark the answers with an X)**

DIMENSIONS	DECISION MAKING CRITERIA	ACESS
Health System Criteria	Access to the doctor	
	Access to the hospital bed	
	Security agencies support	
Support Tools Criteria	Ambulance location	
	Assistance system access	
	Support material access	
Victims Criteria	Call time	
	Victim location (e.g. home, public places)	
	Proper means of transportation	
	Health insurance	
	Sex	
	Age	
	Willingness	
	Refusal	
Victim's Health Status Criteria	State of consciousness	
	Airway and oxygenation	
	Pulse	
	Traumatization	
	Victim's health history	
	Alcohol or drug intake	

External Factors Criteria	Social commotion	
	Heavy traffic	
	Bad weather (e.g. rain or strong winds)	
	Insistence of people close to the victim	
	Hard to reach address	

Question 02

At the time of the decision, based on your experience and skills, could any other criterion or dimension not present in the protocols or in this questionnaire be added?
